# Pulmonary vascular distensibility with passive leg raise is comparable to exercise and predictive of clinical outcomes in pulmonary hypertension

**DOI:** 10.1002/pul2.12029

**Published:** 2022-01-12

**Authors:** Callyn J. Kozitza, Naga Dharmavaram, Ran Tao, Diana M. Tabima, Naomi C. Chesler, Farhan Raza

**Affiliations:** ^1^ Department of Biomedical Engineering Madison Wisconsin USA; ^2^ Department of Medicine Cardiovascular Division Madison Wisconsin USA; ^3^ Department of Medicine University of Wisconsin‐Madison Madison Wisconsin USA; ^4^ Department of Biomedical Engineering, Edwards Lifesciences Foundation Cardiovascular Innovation and Research Center University of California, Irvine Irvine California USA

**Keywords:** invasive cardiopulmonary exercise test, passive leg raise, pulmonary hypertension, pulmonary vascular distensibility

## Abstract

Pulmonary vascular distensibility (*α*) is a marker of the ability of the pulmonary vasculature to dilate in response to increases in cardiac output, which protects the right ventricle from excessive increases in afterload. α measured with exercise predicts clinical outcomes in pulmonary hypertension (PH) and heart failure. In this study, we aim to determine if α measured with a passive leg raise (PLR) maneuver is comparable to α with exercise. Invasive cardiopulmonary exercise testing (iCPET) was performed with hemodynamics recorded at three stages: rest, PLR and peak exercise. Four hemodynamic phenotypes were identified (2019 ECS guidelines): pulmonary arterial hypertension (PAH) (*n* = 10), isolated post‐capillary (Ipc‐PH) (*n* = 18), combined pre‐/post‐capillary PH (Cpc‐PH) (*n* = 15), and Control (no significant PH at rest and exercise) (*n* = 7). Measurements of mean pulmonary artery pressure, pulmonary artery wedge pressure, and cardiac output at each stage were used to calculate α. There was no statistical difference between α‐exercise and α‐PLR (0.87 ± 0.68 and 0.78 ± 0.47% per mmHg, respectively). The peak exercise‐ and PLR‐based calculations of α among the four hemodynamic groups were: Ipc‐PH = Ex: 0.94 ± 0.30, PLR: 1.00 ± 0.27% per mmHg; Cpc‐PH = Ex: 0.51 ± 0.15, PLR: 0.47 ± 0.18% per mmHg; PAH = Ex: 0.39 ± 0.23, PLR: 0.34 ± 0.18% per mmHg; and the Control group: Ex: 2.13 ± 0.91, PLR: 1.45 ± 0.49% per mmHg. Patients with *α* ≥ 0.7% per mmHg had reduced cardiovascular death and hospital admissions at 12‐month follow‐up. In conclusion, α‐PLR is feasible and may be equally predictive of clinical outcomes as α‐exercise in patients who are unable to exercise or in programs lacking iCPET facilities.

## INTRODUCTION

The ability of the right ventricle (RV) to adapt to increased afterload by the pulmonary circulation defines clinical outcomes in pulmonary hypertension (PH).^1^, ^2^ Hemodynamically, PH is classified into three groups: pre‐capillary, isolated post‐capillary (Ipc‐PH), and combined pre‐/post‐capillary PH (Cpc‐PH).^3^, ^4^ Based on clinical phenotypes, PH is classified into five World Health Organization (WHO) groups.^3^ Among all PH phenotypes, the increased RV afterload is a unifying feature, which influences ventricular‐vascular coupling and RV function.^5^, ^6^ Early identification of increased RV afterload with provocative maneuvers can lead to prompt and focused interventions.^7^, ^8^, ^9^

RV afterload has two main components: (1) a static (mean) component described by pulmonary vascular resistance (PVR), and (2) an unsteady (pulsatile) component dependent on proximal artery compliance (PCa) and distal arteriolar distensibility (α). Distensibility is defined as the percent increase in diameter (or area) of the smallest pulmonary arteries per mmHg increase in blood pressure. The ability of the pulmonary vasculature to dilate helps to protect the RV from excessive increases in pressure with increases in cardiac output (CO). In healthy individuals, the increase in diameter is 1.5%–2% per mmHg^10^, ^11^ which helps to maintain a low PVR during exercise.^12^ Reduced distensibility has been previously shown in settings of chronic hypoxia^10^, ^13^ and PH^14^, ^15^, ^16^, ^17^ which can impair exercise capacity and contribute to increased RV afterload.

Linehan et al.^18^ developed a distensible vessel model to predict distensibility with pressure changes for increasing flow. Distensibility is commonly determined using multipoint pressure‐flow data^18^ collected during invasive cardiopulmonary exercise testing (iCPET). However, patients with PH may not be able or willing to exercise. A passive leg raise (PLR) is a simple maneuver that shifts approximately 300 ml of blood from the venous system of the lower extremities back toward the heart, resulting in an increase in preload and pulmonary capillary wedge pressure (PCWP).^19^ Here, we sought to determine whether distensibility calculated with a PLR maneuver was comparable to distensibility calculated with exercise in three different phenotypes of PH (pulmonary arterial hypertension: PAH, Ipc‐PH, and Cpc‐PH) and a control group (with no significant PH at rest and exercise).

## METHODS

### Study population

The study cohort included subjects presenting to our University PH clinic for undefined dyspnea and referred for an iCPET for clinical indication for dyspnea workup. We evaluated 50 consecutive subjects over approximately a 1‐year period: December 2019–January 2021. Per 2019 ESC/ERS guidelines, PH diagnosis was based on mean pulmonary artery pressure (mPAP) >20 mmHg at rest. Study subjects were further classified into one of three PH phenotypes: PAH (PCWP ≤ 15 mmHg and PVR ≥ 3 WU), Ipc‐PH (PCWP > 15 mmHg, and PVR < 3 WU), Cpc‐PH (PCWP > 15 mmHg, and PVR ≥ 3 WU).^3^ PVR was calculated as

(1) $$\text{PVR}=\frac{\text{mPAP}-\text{PCWP}}{\text{CO}}.$$

Subjects with mPAP ≤ 20 mmHg at rest, mPAP/CO slope with exercise < 3.0 mmHg/L⋅min^−1^ and exercise total pulmonary resistance (TPR) < 3.0 Wood units were placed in the Control group.^20^, ^21^ Exclusion criteria included: need for supplemental oxygen or reported desaturation (SpO_2_ ≤ 95%) on 6‐min walk test (before iCPET), serum creatinine >2 mg/dl or requiring dialysis, pulmonary veno‐occlusive disease, abdominal compressions, or lymphedema.

### Invasive cardiopulmonary exercise testing

All subjects underwent invasive CPET with simultaneous right heart catheterization and expired gas analysis (Metabolic Ultima™ CardiO2®; MedGraphics) at rest, PLR, and during exercise. Right internal jugular venous access was obtained with a 7‐Fr venous sheath and a balloon‐tipped, double‐lumen, fluid‐filled 7‐Fr pulmonary artery (PA) catheter. The metabolic cart was connected to the subjects via a mouthpiece and a nose‐clip was placed to avoid any air leak. At rest, right atrial pressure (RAP), RV pressure, pulmonary artery pressure (PAP), PCWP and CO were recorded at end‐expiration with the subject in a supine position. CO was measured via both direct Fick principle (oxygen consumption: VO_2_ recorded at the time of mixed venous sample collection) and thermodilution methods.

Next, the subject's legs were placed in the pedals of a stationary supine ergometer (Lode Angio‐imaging with fixation set for instrument rails: part number 967905) with thighs at a 90° angle to the abdomen and the knees were bent. The subject's upper body was lifted to 45° incline with a firm, wedge‐shaped pillow. At this time, the pressure transducer connected to the PA catheter was adjusted in this semi‐recumbent position to the level of the right atrium. After 5 min, repeat hemodynamics were recorded for the PLR stage. Subsequently, exercise was started with unloaded peddling (0 W) for 2 min and then resistance was increased on an incremental 10–20 W/min protocol. Thermodilution CO was performed at every 25 Watts. This was a symptom limiting test, and all subjects were exercised to a maximum tolerated stage until they requested to stop the test due to significant dyspnea and/or muscle fatigue. The mixed venous sample was drawn near peak exercise with the subject still pedaling (with VO_2_ noted for direct Fick). Repeat hemodynamics were recorded at peak exercise in this order: PAP, PCWP, RAP (via side port) and then RV pressure. The PA catheter and venous sheath were removed at the end of the study. No radial arterial line was used for patient comfort. However, all subjects underwent a pre‐test (iCPET) 6‐min walk test and maintained pulse oximetry >95%.

### Distensibility and compliance

End‐expiratory hemodynamic measurements of mPAP, PCWP, and CO at rest, PLR and peak exercise were utilized in the distensible vessel model.^18^ mPAP was calculated as a weighted time average over three cardiac cycles, as previously reported.^22^ Assuming that the pulmonary arteries are fully recruited, dilated and homogeneously distensible, α can be calculated using the equation:^18^

(2) $$\text{mPAP}=\frac{{\left[{\left(1+\alpha \text{PCWP}\right)}^{5}+5\alpha \left(\text{TPR}\right)\text{CO}\right]}^{\frac{1}{5}}-1}{\alpha },$$

where TPR is the total pulmonary resistance at rest, calculated as

(3) $$\text{TPR}=\frac{\text{mPAP}}{\text{CO}}.$$

Using the method of successive iterations,^18^ α‐exercise was fit to rest and peak exercise; α‐PLR was fit to rest and PLR. Fitting was done with a custom Matlab code identical to that used by Argiento et al.^16^  and Chesler et al.^23^

Large artery compliance (PCa) was calculated as the ratio of stroke volume (SV) to pulmonary artery pulse pressure (PP). SV was computed as CO divided by heart rate (HR).

### Statistical analysis

All results are presented as mean ± standard deviation and *p* value < 0.05 was considered significant. Results were analyzed for condition (peak exercise vs. PLR) and hemodynamic group using a multiple linear regression model, with repeated measures, and with PLR and hemodynamic groups as fixed effects. Model assumptions were verified by examining model diagnostic plots. Tukey's honestly significant difference test was used as a post hoc test of significance. Categorical data were analyzed using Fischer's exact test. The prognostic value of pulmonary vascular distensibility to predict cardiovascular death and hospitalization in PH subjects was determined using Kaplan–Meier survival with Log‐Rank testing. All statistical analyses were conducted using GraphPad Prism version 8.3.1 (GraphPad Software, Inc.), R (version 4.0.3), and JMP Pro (Version 15).

## RESULTS

### Clinical characteristics

Baseline characteristics of the four hemodynamic phenotypes are shown in Table 1. The mean age of the entire cohort (*n* = 50) was 65 ± 15 years. The subjects in the Control group were younger (47 ± 15 years). Overall, 46% of the subjects were female, with similar predominance across the four groups. Subjects presented to the clinic with exertional dyspnea and median New York Health Association (NYHA) class 3 (interquatile range [IQR]: 2–3). PAH subjects (*n* = 10) included WHO Group I (*n *= 7; two with idiopathic PAH, four with connective tissue disease and one with portal hypertension), WHO Group III (*n* = 2), or WHO Group IV (*n* = 1). PH vasodilator therapies were initiated in all PAH subjects (after the iCPET), and none of the subjects in rest of the cohort.

**Table 1:** Clinical characteristics of the four subject groups

PH phenotype	Control (*n* = 7)	Ipc‐PH (*n* = 18)	Cpc‐PH (*n* = 15)	PAH (*n* = 10)
Age, years	47 ± 15	69 ± 12*	65 ± 15*	71 ± 9*
Sex, female, *n* (%)^a^	4 (57)	8 (44)	6 (40)	5 (50)
BMI (kg/m^2^)	26 ± 4	34 ± 9	32 ± 7	25 ± 6#
6MWD (m)	428 ± 9	247 ± 84	235 ± 95	309 ± 110
BNP (pg/ml)	47 ± 46	294 ± 291	310 ± 326	341 ± 284
Creatinine (mg/dl)	0.9 ± 0.3	1.2 ± 0.4	1.4 ± 0.5	1.0 ± 0.3
Medications, *n* (%)^a^				
Diuretics	3 (43)	16 (89)	14 (93)	8 (80)
Anticoagulants	1 (14)	11 (61)	5 (33)	2 (20)
Comorbidities, *n* (%)^a^				
Diabetes mellitus	1 (14)	8 (44)	1 (7)	3 (30)
Hypertension§	2 (29)	14 (78)	13 (87)	6 (60)
Chronic kidney disease	1 (14)	7 (39)	9 (60)	2 (20)
Atrial fibrillation	1 (14)	10 (56)	6 (40)	1 (10)
Obstructive sleep apnea	1 (14)	10 (56)	7 (47)	2 (20)
Coronary artery disease	0 (0)	9 (50)	7 (47)	2 (20)
Chronic obstructive pulmonary disease	2 (29)	4 (22)	3 (20)	1 (10)
Pulmonary embolism history	1 (14)	3 (17)	1 (7)	2 (20)
NYHA Functional Class, median (IQR)	2 (0)	3 (1)	3 (1)	3 (1)

*Note*: *p* < 0.05 versus Cpc‐PH within each condition.

Abbreviations: 6MWD, 6‐min walk distance; BMI, body mass index; BNP, brain natriuretic peptide; Cpc‐PH, combined pre‐/post‐capillary PH; Ipc‐PH, isolated post‐capillary; IQR, interquartile range; NYHA, New York heart association; PAH, pulmonary arterial hypertension; PH, pulmonary hypertension.

^a^ Fisher's exact test used for categorical data.

* *p* < 0.05 versus no PH.

^#^ *p* < 0.05 versus Ipc‐PH.

^§^ *p* < 0.05 no PH versus Ipc‐PH versus Cpc‐PH versus PAH.

### Invasive hemodynamics and pulmonary vascular distensibility

**Figure 1:**
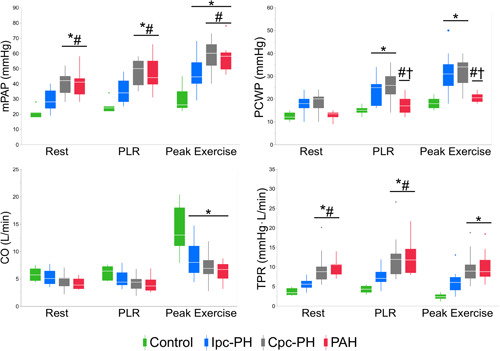
Hemodynamic measurements at rest, PLR and peak exercise for the four groups: Control (*n* = 7, green), Ipc‐PH (*n* = 18, blue), Cpc‐PH (*n* = 15, grey), and PAH (n = 10, red). *p < 0.05 versus Control; ^#^ *p* < 0.05 versus Ipc‐PH; †*p* < 0.05 versus Cpc‐PH within each condition. CO, cardiac output; Cpc‐PH, combined pre/post‐capillary PH; Ipc‐PH, isolated post‐capillary PH; mPAP, mean pulmonary artery pressure; PAH, pulmonary arterial hypertension; PCWP, pulmonary capillary wedge pressure; PLR, passive leg raise maneuver; TPR, total pulmonary resistance

Table 2 presents the hemodynamic measurements collected at rest, PLR, and peak exercise in each group. As expected, PCWP was highest in the Ipc‐PH and Cpc‐PH groups based on the subject classification. While the mPAP increased (with PLR and exercise) among all subjects, the control group had a 2.5‐times augmentation of cardiac output with exercise, while rest of the cohort (different PH phenotypes) were unable to increase their cardiac output by two‐fold. This resulted in preserved compliance (PCa) in the control group, and reduced PCa among rest of the cohort (Table 2). Distensibility was determined using measurements of mPAP, PCWP, CO, and TPR (Figure 1).

**Table 2:** Hemodynamic measurements from iCPET

PH phenotype	Control (*n* = 7)	Ipc‐PH (*n* = 18)	Cpc‐PH (*n* = 15)	PAH (*n* = 10)
mPAP (mmHg)				
Rest	21 ± 4	29 ± 7	41 ± 7*, #	40 ± 9*, #
PLR	25 ± 4	35 ± 7	48 ± 8*, #	47 ± 10*, #
Peak exercise	30 ± 8	46 ± 10*	59 ± 8*, #	58 ± 9*, #
PCWP (mmHg)				
Rest	13 ± 2	17 ± 4	18 ± 4	13 ± 2
PLR	15 ± 2	24 ± 6*	26 ± 5*	17 ± 4#, †
Peak exercise	18 ± 2	31 ± 7*	32 ± 6*	21 ± 2#, †
CO (L/min)				
Rest	5.9 ± 1.1	5.3 ± 1.4	4.7 ± 1.2	4.0 ± 1.0
PLR	6.1 ± 1.2	5.0 ± 1.4	4.3 ± 1.2	9.0 ± 1.3
Peak exercise	13.9 ± 4.3	8.5 ± 3.0*	7.0 ± 2.2*	6.4 ± 1.7*
CI (L/min/m^2^)				
Rest	3.1 ± 0.6	2.5 ± 0.5	2.4 ± 0.5	2.2 ± 0.4
PLR	3.2 ± 0.7	2.3 ± 0.5	2.2 ± 0.5	2.2 ± 0.5
Exercise	7.3 ± 2.3	3.9 ± 1.1*	3.5 ± 1.0*	3.5 ± 0.7*
SVI (ml/m^2^)				
Rest	40 ± 8	37 ± 10	33 ± 8	35 ± 9
PLR	41 ± 10	35 ± 10	30 ± 7	32 ± 5
Peak exercise	53 ± 12	43 ± 11	38 ± 10*	38 ± 8
PVR (wood units)				
Rest	1.4 ± 0.7	2.2 ± 0.6	5.0 ± 1.9*, #	6.6 ± 1.8*, #
PLR	1.6 ± 0.6	2.4 ± 0.8	5.5 ± 2.0*, #	7.7 ± 2.7*, #, †
Peak exercise	0.8 ± 0.6	2.0 ± 0.7	4.1 ± 1.4*, #	6.4 ± 2.6*, #, †
DPG (mmHg)				
Rest	3 ± 3	5 ± 4	11 ± 5	14 ± 7*, #
PLR	4 ± 4	3 ± 3	8 ± 4	15 ± 9*, #
Peak exercise	5 ± 6	4 ± 4	10 ± 7	19 ± 9*, #, †
HR (bpm)				
Rest	78 ± 11	67 ± 10	74 ± 12	66 ± 8
PLR	80 ± 7	68 ± 12	75 ± 13	68 ± 7
Peak exercise	136 ± 18	91 ± 20*	96 ± 20*	91 ± 14*
PCa (ml/mmHg)				
Rest	6.1 ± 1.8	4.6 ± 1.5	2.4 ± 0.9*, #	1.9 ± 0.5*, #
PLR	5.8 ± 1.6	3.7 ± 1.5*	1.7 ± 0.6*, #	1.6 ± 0.4*, #
Peak exercise	4.5 ± 1.8	2.9 ± 1.0	1.5 ± 0.5*, #	1.5 ± 0.7*
Hematocrit (%)				
Rest	38 ± 5	38 ± 6	40 ± 5	42 ± 7
PLR	39 ± 5	38 ± 5	40 ± 4	43 ± 7
Peak exercise	41 ± 6	40 ± 6	41 ± 5	44 ± 8
Peak workload parameters				
Peak exercise TPR (Wood units)	2.2 ± 0.6	6.1 ± 2.4	9.4 ± 3.7*	10.0 ± 3.9*
mPAP/CO slope (mmHg/L/min)	1.1 ± 0.7	17.6 ± 44.4	11.8 ± 7.3	27.1 ± 41.2
Workload (W)	131 ± 68	64 ± 26*	45 ± 28*	40 ± 26*
% age‐predicted	83 ± 42	58 ± 21	37 ± 22*	48 ± 29
VO_2_ (ml/kg/min)	17.7 ± 8.7	9.0 ± 2.3*	8.5 ± 2.7*	9.6 ± 2.8*
% age‐predicted	62 ± 29	44 ± 12	38 ± 14*	43 ± 14
O_2_ pulse (ml/min)	9.7 ± 3.4	10.0 ± 2.3	7.7 ± 2.4	7.1 ± 1.4
% age‐predicted	72 ± 29	44 ± 12	38 ± 14	43 ± 14
ETCO_2_ (mmHg)	35 ± 6	32 ± 5	28 ± 6*	26 ± 4*
V_E_/VO_2_	35 ± 5	39 ± 6	45 ± 12	46 ± 11

Abbreviations: CI, cardiac index; CO, cardiac output; Cpc‐PH, combined pre‐/post‐capillary PH; DPG, diastolic pressure gradient; ETCO_2_, end‐tidal carbon dioxide; HR, heart rate; iCPET, invasive cardiopulmonary exercise test; Ipc‐PH, isolated post‐capillary; mPAP, mean pulmonary artery pressure; O_2_ pulse, VO_2_/HR; PAH, pulmonary arterial hypertension; PCa, pulmonary vascular compliance; PCWP, pulmonary capillary wedge pressure; PH, pulmonary hypertension; PLR, passive leg raise; PVR, pulmonary vascular resistance; SV, stroke volume; SVI, stroke volume index; TPR, total pulmonary resistance; VE/VO_2_, minute ventilation to carbon dioxide production slope; VO_2_, oxygen consumption.

* *p* < 0.05 versus no PH.

^#^ *p* < 0.05 versus Ipc‐PH.

^†^ *p* < 0.05 versus Cpc‐PH within each condition.

**Figure 2:**
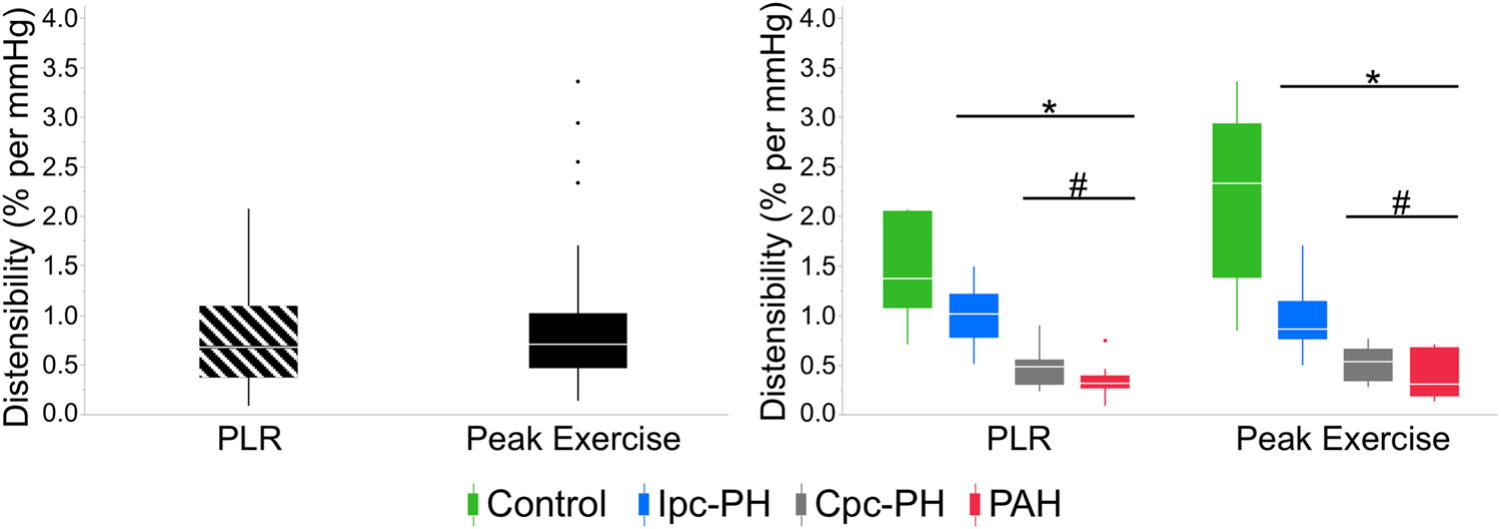
Distensibility calculated from PLR (0.78 ± 0.47% per mmHg) and peak exercise (0.81 ± 0.54% per mmHg) with no statistically significant difference, *p* = 0.263 (left). Significant differences between the four groups were identical with distensibility based on PLR‐ and peak exercise‐based calculations: Control (*n* = 7, green), Ipc‐PH (*n* = 18, blue), Cpc‐PH (*n* = 15, gray), and PAH (*n* = 10, red) (right). *p < 0.05 versus Control; ^#^ *p* < 0.05 versus Ipc‐PH; †*p* < 0.05 versus Cpc‐PH within each condition. Cpc‐PH, combined pre/post‐capillary PH; Ipc‐PH, isolated post‐capillary PH; PAH, pulmonary arterial hypertension; PLR, passive leg raise maneuver

**Figure 3:**
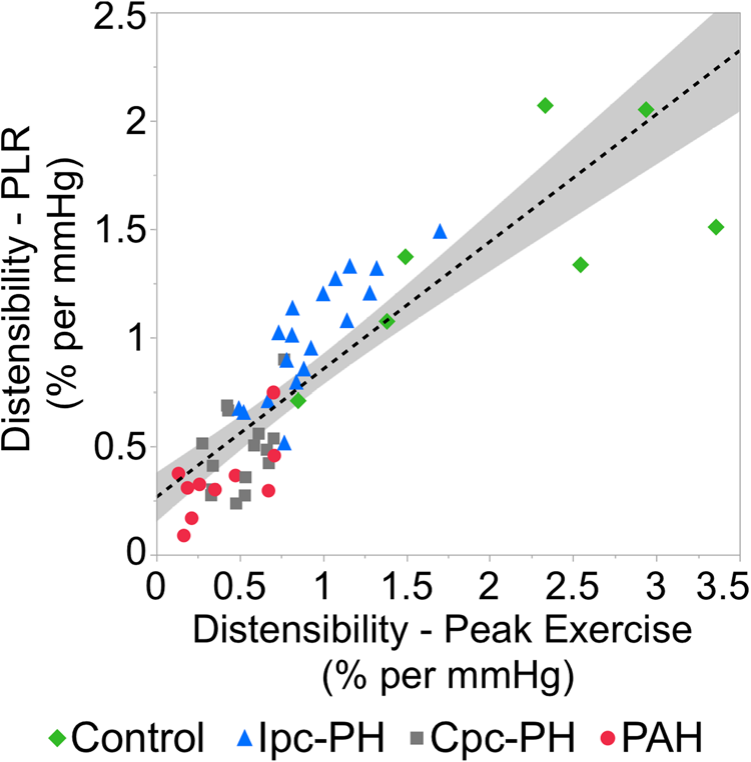
Distensibility with exercise is linearly correlated with distensibility with PLR (*p* < 0.001, adjusted *R* ^2^ = 0.73). PLR, passive leg raise maneuver

Among the overall cohort, there was no statistically significant difference (*p* = 0.200) between the two measurements of distensibility: peak exercise and PLR (Figure 2). The peak exercise‐ and PLR‐based measures of distensibility were significantly reduced in the Ipc‐PH, Cpc‐PH, and PAH groups compared to the Control group (Figure 2). Additionally, distensibility was lower in the groups with a pre‐capillary PH component compared to the Control and Ipc‐PH groups (Figure 2). As represented in Figure 3, distensibility with exercise was linearly correlated with distensibility with PLR among the overall cohort (adjusted *R* ^2^ = 0.73, *p* < 0.001). Taking four hemodynamic groups into account (as a fixed categorical variable, without interaction) further improves the model fit (adjusted *R* ^2^ = 0.83, *p* < 0.001). The intercepts were different among the four hemodynamics groups (intercepts for Control = 0.11, Ipc‐PH= 0.45, Cpc‐PH = 0.17, and PAH = 0.43).

Since the distensible vessel model assumes constant hematocrit,^18^ serial hemoglobin (Hgb) measurements were obtained. Hematocrit was computed from Hgb using a Hct (%)/Hgb (g/dl) ratio of 3 (Table 2). There was no difference in Hct (*p* = 0.234) between conditions (rest vs. PLR vs. peak exercise).

### Prognostic value of pulmonary vascular distensibility

**Figure 4:**
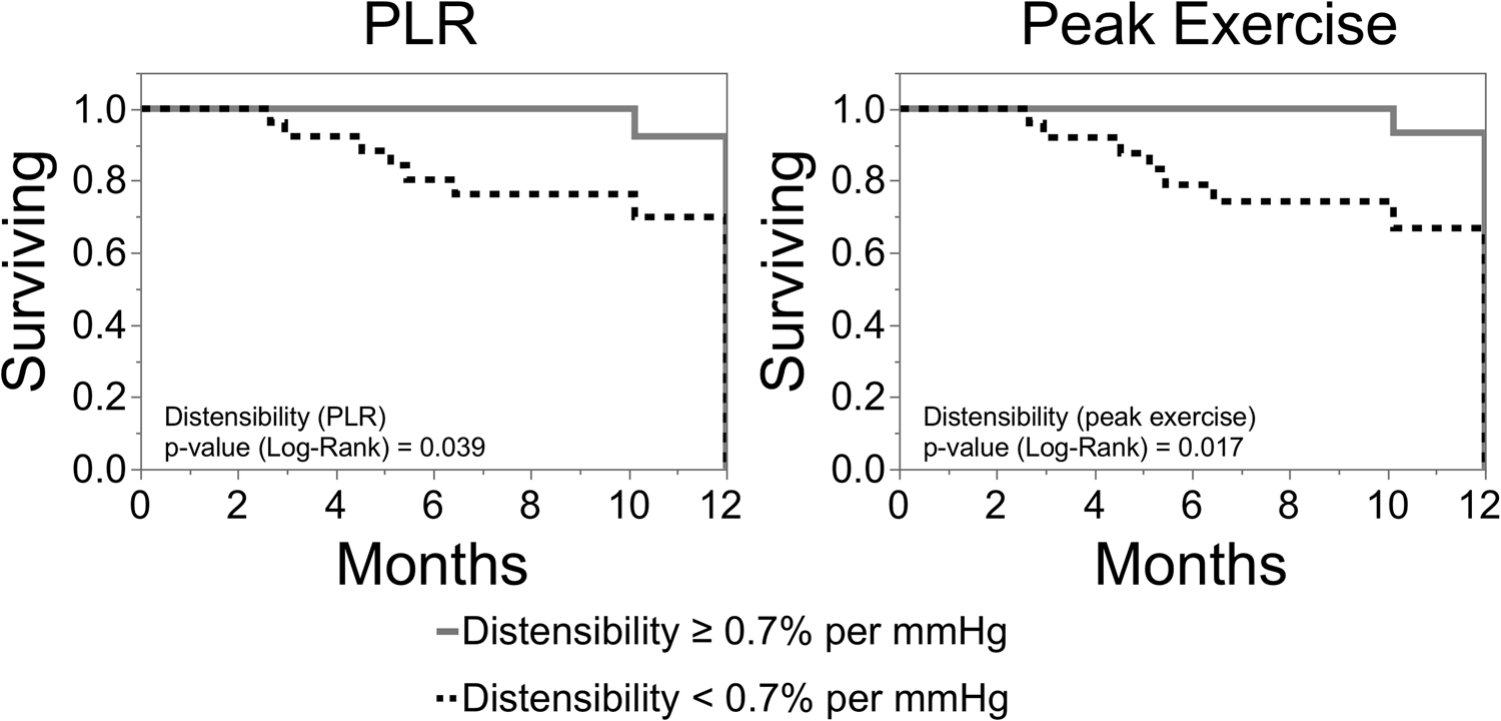
Survival from cardiovascular death and hospitalization based on distensibility with PLR maneuver (left) was reportable, and similar to distensibility with exercise (right). PLR, passive leg raise maneuver

Distensibility is known to predict cardiovascular outcomes in pulmonary hypertension and heart failure.^14^, ^24^ To evaluate survival from adverse outcomes (defined as cardiovascular death and hospital admissions) within one‐year after the exercise study, we dichotomized distensibility based on previously published historical data (<0.7% and ≥0.7% per mmHg).^14^ This was consistent with the median value of our study cohort (0.82% per mmHg). Kaplan–Meier time‐to‐event survival analysis revealed that subjects with distensibility ≥0.7% per mmHg had reduced cardiovascular death and hospital admissions (Figure 4).

Among the overall cohort (*n* = 50), the median follow‐up was 12‐months. The event rate and median survival were similar among distensibility values assessed with PLR and exercise. Among subjects with distensibility <0.7% per mmHg, two subjects died and five had hospital admission from cardiovascular cause. Among subjects with distensibility ≥0.7% per mmHg, zero subjects died and one subject had a cardiovascular hospital admission. The median survival from adverse outcomes was 10.2 months for distensibility <0.7% per mmHg and 11.9 months for distensibility ≥0.7% per mmHg.

## DISCUSSION

Right ventricular function is a key determinant in the clinical presentation and prognosis of pulmonary hypertension. RV function in turn, depends on RV afterload. Increased stiffness of the large pulmonary arteries is associated with worse RV performance.^25^ An increase in stiffness (decrease in PCa) was observed in all PH groups compared to the Control group with PLR and at peak exercise (Table 2). Loss of distal arteriolar distensibility also contributes to increases in RV afterload since α acts to limit the increase in RV afterload in disease and with exercise. Using exercise to measure α, reduced distensibility predicts exercise capacity and survival in left heart failure.^14^ However, many subjects with PH are not able to exercise. Using the distensible vessel model,^18^ we have shown that in our cohort of subjects with and without PH, pulmonary vascular distensibility can be reliably measured via PLR, which provides prognostic value similar to distensibility with exercise.

The distensibility with exercise measured here was similar to previously reported values in healthy subjects and those with different PH phenotypes. Present calculations of distensibility (with exercise) for the Control group agree with prior studies in which a value of 1.5%–2% per mmHg was observed.^10^, ^11^ Distensibility with exercise was decreased in all PH phenotypes, with the lowest values obtained in the groups with a pre‐capillary component (Cpc‐PH and PAH). These results are also comparable to previously reported distensibility values in the range of 0.25%–0.4% per mmHg in PAH, and around 0.8%–0.9% per mmHg in heart failure with either reduced or preserved ejection fraction.^14^ The Cpc‐PH group had a calculated distensibility closer to that of the PAH group than the Ipc‐PH group. This finding is consistent with previous studies demonstrating that the PVC‐PVR relationship in Cpc‐PH more closely resembles PAH than Ipc‐PH.^26^

We also sought to determine whether distensibility measured with a passive leg raise maneuver was comparable to distensibility measured with exercise. We note that our PLR methodology was in semi‐recumbent position, which is different than a pure supine position of previously reported PLR studies by Borlaug et al.^8^ The semi‐recumbent position was chosen for patient comfort and to allow longer duration of exercise. Among the Ipc‐PH and Cpc‐PH subjects in our study, the observations of PCWP > 20 mmHg with PLR along with a 7–8‐point increase in PCWP from rest‐to‐PLR (Table 2), indicates a physiologically significant increased venous return. This behavior of PCWP with PLR in post‐capillary PH in our study is also consistent with the HELP trial.^9^ In the HELP trial, a PCWP > 20 mmHg with PLR maneuver was an inclusionary criteria that qualifies a subject with diagnosis of PH due to heart failure with preserved ejection fraction. Based on this methodology, distensibility was also calculated with PLR. Among the overall cohort, there was no statistical difference between α‐exercise and α‐PLR (Figure 2) and there was good correlation between α‐exercise and α‐PLR (Figure 3). We interpret the lack of difference as an indication that the response of the small arterial pulmonary circulation (which is reflected by the metric of distensibility) to slight volume loading by PLR is equivalent to the response to much greater volume loading by exercise.

While exercise is a physiological stressor that increases blood flow resulting in an increase in mPAP,^20^ we noted a similar response with increased preload due to the PLR maneuver. In the Control group, increases in flow were accompanied by small increases in pressure indicating high distensibility. The Ipc‐PH group had small increases in flow with large increases in pressure. Finally, the Cpc‐PH and PAH groups had smallest increases in flow combined with the largest increases in pressure, resulting in significantly lower calculated distensibility (Figure 2).

Lastly, the prognostic value of distensibility with PLR was similar to that of distensibility with exercise (Figure 4). Using a previously validated cut‐off of distensibility (0.7% per mmHg with exercise), the survival from cardiovascular death and hospitalization based on α‐PLR was reportable, and comparable to, α‐exercise. This adds to the value of PLR in discriminating different disease phenotypes and acquiring prognostic information in different PH phenotypes.

Several limitations of the study should be noted. All measurements were collected from a single‐center cohort of subjects with a moderate sample size (*n *= 50). While a moderate sample size may lead to a type I error, a strong correlation of distensibility with PLR and exercise (Figure 3), along with a sound physiological interpretation, provides confidence in the results. CPET procedures are not commonly performed in subjects without significant cardiopulmonary disease; all subjects in the Control group were undergoing testing for dyspnea and suspicion of PH and may not be representative of a completely healthy population. This may result in the underestimation of the differences between the PH groups and healthy controls. Due to the size of the study population (*n* = 50), data were not disaggregated or analyzed for sex differences. In healthy subjects, pulmonary vascular distensibility has been shown to decrease with age.^10^, ^17^ Sex differences have also been observed in healthy subjects, with greater pulmonary vascular distensibility observed in women <50 years old.^17^ Lastly, a radial arterial line was not used which limits assessment of mechanism of dead space ventilation due to unavailability of parameters including partial pressure of carbon dioxide. Given the focus of our study was on invasive hemodynamics, the results and interpretations were not affected.

In summary, this study shows that pulmonary vascular distensibility can be measured via PLR and may provide prognostic information similar to distensibility with exercise. This finding is clinically important for subjects who are unable to exercise and/or for clinicians who do not have exercise cardiac catheterization facilities. While this study reports on subjects with NYHA II‐III, future studies are needed to validate these findings on a larger scale and different disease phenotypes and severity.

## CONFLICT OF INTERESTS

The authors declare that there are no conflict of interests.

## ETHICS STATEMENT

The study was approved by the UW‐Madison institutional review board (UW‐IRB ID: 2019‐0535). The IRB waived the participant consent due to retrospective nature of the study, whereas the protected health information was kept anonymous except for the corresponding author. The study complies with the guidelines of Declaration of Helsinki.

## AUTHOR CONTRIBUTIONS

Farhan Raza and Naga Dharmavaram collected the data. Callyn J. Kozitza, Farhan Raza, Naomi C. Chesler reviewed the data, performed analysis and formulated the manuscript. Callyn J. Kozitza, Farhan Raza and Naomi C. Chesler wrote the manuscript. All co‐authors reviewed and approved the manuscript before submission.

## References

[1] Vanderpool RR , Pinsky MR , Naeije R , Deible C , Kosaraju V , Bunner C , Mathier MA , Lacomis J , Champion HC , Simon MA . RV‐pulmonary arterial coupling predicts outcome in patients referred for pulmonary hypertension. Heart. 2015;101:37–43. 10.1136/heartjnl-2014-306142 25214501 PMC4268056

[2] Tello K , Dalmer A , Axmann J , Vanderpool R , Ghofrani HA , Naeije R , Roller F , Seeger W , Sommer N , Wilhelm J , Gall H , Richter MJ . Reserve of right ventricular‐arterial coupling in the setting of chronic overload. Circ Heart Fail. 2019;12:005512. 10.1161/CIRCHEARTFAILURE.118.005512 30616360

[3] Simonneau G , Montani D , Celermajer DS , Denton CP , Gatzoulis MA , Krowka M , Williams PG , Souza R . Haemodynamic definitions and updated clinical classification of pulmonary hypertension. Eur Respir J. 2019;53:1801913. 10.1183/13993003.01913-2018 PMC6351336 30545968

[4] Al‐Omary MS , Sugito S , Boyle AJ , Sverdlov AL , Collins NJ . Pulmonary hypertension due to left heart disease: diagnosis, pathophysiology, and therapy. Hypertension. 2020;75:1397–1408. 32336230 10.1161/HYPERTENSIONAHA.119.14330

[5] Hsu S , Houston BA , Tampakakis E , Bacher AC , Rhodes PS , Mathai SC , Damico RL , Kolb TM , Hummers LK , Shah AA , McMahan Z , Corona‐Villalobos CP , Zimmerman SL , Wigley FM , Hassoun PM , Kass DA , Tedford RJ . Right ventricular functional reserve in pulmonary arterial hypertension. Circulation. 2016;133:2413–22. 27169739 10.1161/CIRCULATIONAHA.116.022082 PMC4907868

[6] Guazzi M , Dixon D , Labate V , Beussink‐Nelson L , Bandera F , Cuttica MJ , Shah SJ . RV contractile function and its coupling to pulmonary circulation in heart failure with preserved ejection fraction: stratification of clinical phenotypes and outcomes. JACC Cardiovasc Imaging. 2017;10:1211–21. 10.1016/j.jcmg.2016.12.024 28412423

[7] Andersen MJ , Olson TP , Melenovsky V , Kane GC , Borlaug BA . Differential hemodynamic effects of exercise and volume expansion in people with and without heart failure. Circ Heart Fail. 2015;8:41–8. 25342738 10.1161/CIRCHEARTFAILURE.114.001731

[8] Borlaug BA . Exercise haemodynamics and outcome in patients with dyspnoea. Eur Heart J. 2014;35:3085–87. 25161179 10.1093/eurheartj/ehu350

[9] Burkhoff D , Borlaug BA , Shah SJ , Zolty R , Tedford RJ , Thenappan T , Zamanian RT , Mazurek JA , Rich JD , Simon MA , Chung ES , Raza F , Majure DT , Lewis GD , Preston IR , Rich S . Levosimendan improves hemodynamics and exercise tolerance in PH‐HFpEF: results of the randomized placebo‐controlled HELP trial. JACC Heart Fail. 2021;9:360–70. 33839076 10.1016/j.jchf.2021.01.015

[10] Reeves JT , Linehan JH , Stenmark KR . Distensibility of the normal human lung circulation during exercise. Am J Physiol Lung Cell Mol Physiol. 2005;288:419–25. 10.1152/ajplung.00162.2004 15695542

[11] Lalande S , Yerly P , Faoro V , Naeije R . Pulmonary vascular distensibility predicts aerobic capacity in healthy individuals. J Physiol. 2012;590:4279–88. 22733662 10.1113/jphysiol.2012.234310 PMC3473285

[12] Naeije R , Vanderpool R , Dhakal BP , Saggar R , Saggar R , Vachiery JL , Lewis GD . Exercise‐induced pulmonary hypertension: physiological basis and methodological concerns. Am J Respir Crit Care Med. 2013;187:576–83. 23348976 10.1164/rccm.201211-2090CI PMC3733438

[13] Mulchrone A , Moulton H , Eldridge MW , Chesler NC . Susceptibility to high‐altitude pulmonary edema is associated with increased pulmonary arterial stiffness during exercise. J Appl Physiol. 2020;128:514–22. 31854245 10.1152/japplphysiol.00153.2019 PMC7099440

[14] Malhotra R , Dhakal BP , Eisman AS , Pappagianopoulos PP , Dress A , Weiner RB , Baggish AL , Semigran MJ , Lewis GD . Pulmonary vascular distensibility predicts pulmonary hypertension severity, exercise capacity, and survival in heart failure. Circ Heart Fail. 2016;9:e003011. 10.1161/CIRCHEARTFAILURE.115.003011 27301469 PMC4911900

[15] Blyth KG , Syyed R , Chalmers J , Foster JE , Saba T , Naeije R , Melot C , Peacock AJ . Pulmonary arterial pulse pressure and mortality in pulmonary arterial hypertension. Respir Med. 2007;101:2495–501. 17719764 10.1016/j.rmed.2007.07.004

[16] Argiento P , Chesler N , Mulè M , D'Alto M , Bossone E , Unger P , Naeije R . Exercise stress echocardiography for the study of the pulmonary circulation. Eur Respir J. 2010;35:1273–78. 19926746 10.1183/09031936.00076009 PMC2879460

[17] Argiento P , Vanderpool RR , Mulè M , Russo MG , D'Alto M , Bossone E , Chesler NC , Naeije R . Exercise stress echocardiography of the pulmonary circulation: limits of normal and sex differences. Chest. 2012;142:1158–65. 22539647 10.1378/chest.12-0071 PMC3494470

[18] Linehan JH , Haworth ST , Nelin LD , Krenz GS , Dawson CA. A simple distensible vessel model for interpreting pulmonary vascular pressure‐flow curves. J Appl Physiol. 1992;73(3):987–94. 1400067 10.1152/jappl.1992.73.3.987

[19] Arunachalam A , Chaisson NF , Tonelli AR . Methods to improve the yield of right heart catheterization in pulmonary hypertension. Respir Med X. 2020;2:100015.

[20] Lewis GD , Bossone E , Naeije R , Grünig E , Saggar R , Lancellotti P , Ghio S , Varga J , Rajagopalan S , Oudiz R , Rubenfire M . Pulmonary vascular hemodynamic response to exercise in cardiopulmonary diseases. Circulation. 2013;128:1470–9. 10.1161/CIRCULATIONAHA.112.000667 24060943

[21] Kovacs G , Herve P , Barbera JA , Chaouat A , Chemla D , Condliffe R , Garcia G , Grünig E , Howard L , Humbert M , Lau E , Laveneziana P , Lewis GD , Naeije R , Peacock A , Rosenkranz S , Saggar R , Ulrich S , Vizza D , Vonk Noordegraaf A , Olschewski H . An official European Respiratory Society statement: pulmonary haemodynamics during exercise. Eur Respir J. 2017;50:1700578. 10.1183/13993003.00578-2017 29167297

[22] Mulchrone A , Bellofiore A , Douwes JM , Duong N , Beshish AG , Barton GP , Francois CJ , Eldridge MW , Goss KN , Chesler NC . Impaired right ventricular‐vascular coupling in young adults born preterm. Am J Respir Crit Care Med. 2020;201:615–18. 31697579 10.1164/rccm.201904-0767LE PMC7047464

[23] Chesler NC , Roldan A , Vanderpool RR , Naeije R . How to measure pulmonary vascular and right ventricular function. Annu Int Conf IEEE Eng Med Biol Soc. 2009;2009:177–80. 10.1109/IEMBS.2009.5333835 19964469 PMC3204789

[24] Lau EMT , Chemla D , Godinas L , Zhu K , Sitbon O , Savale L , Montani D , Jaïs X , Celermajer DS , Simonneau G , Humbert M , Hervé P . Loss of vascular distensibility during exercise is an early hemodynamic marker of pulmonary vascular disease. Chest. 2016;149:353–61. 26134583 10.1378/chest.15-0125

[25] Stevens GR , Garcia‐Alvarez A , Sahni S , Garcia MJ , Fuster V , Sanz J . RV dysfunction in pulmonary hypertension is independently related to pulmonary artery stiffness. JACC Cardiovasc Imaging. 2012;5:378–87. 22498327 10.1016/j.jcmg.2011.11.020

[26] Assad TR , Brittain EL , Wells QS , Farber‐Eger EH , Halliday SJ , Doss LN , Xu M , Wang L , Harrell FE , Yu C , Robbins IM , Newman JH , Hemnes AR . Hemodynamic evidence of vascular remodeling in combined post‐ and precapillary pulmonary hypertension. Pulm Circ. 2016;6:313–21. 27683608 10.1086/688516 PMC5019084

